# The Network of Non-coding RNAs in Cancer Drug Resistance

**DOI:** 10.3389/fonc.2018.00327

**Published:** 2018-08-29

**Authors:** Fabio Corrà, Chiara Agnoletto, Linda Minotti, Federica Baldassari, Stefano Volinia

**Affiliations:** Department of Morphology, Surgery and Experimental Medicine, University of Ferrara, Ferrara, Italy

**Keywords:** non-coding RNAs, chemoresistance, drug sensitivity, miRNA, lncRNA, cancer, gene networks

## Abstract

Non-coding RNAs (ncRNAs) have been implicated in most cellular functions. The disruption of their function through somatic mutations, genomic imprinting, transcriptional and post-transcriptional regulation, plays an ever-increasing role in cancer development. ncRNAs, including notorious microRNAs, have been thus proposed to function as tumor suppressors or oncogenes, often in a context-dependent fashion. In parallel, ncRNAs with altered expression in cancer have been reported to exert a key role in determining drug sensitivity or restoring drug responsiveness in resistant cells. Acquisition of resistance to anti-cancer drugs is a major hindrance to effective chemotherapy and is one of the most important causes of relapse and mortality in cancer patients. For these reasons, non-coding RNAs have become recent focuses as prognostic agents and modifiers of chemo-sensitivity. This review starts with a brief outline of the role of most studied non-coding RNAs in cancer and then highlights the modulation of cancer drug resistance via known ncRNAs based mechanisms. We identified from literature 388 ncRNA-drugs interactions and analyzed them using an unsupervised approach. Essentially, we performed a network analysis of the non-coding RNAs with direct relations with cancer drugs. Within such a machine-learning framework we detected the most representative ncRNAs-drug associations and groups. We finally discussed the higher integration of the drug-ncRNA clusters with the goal of disentangling effectors from downstream effects and further clarify the involvement of ncRNAs in the cellular mechanisms underlying resistance to cancer treatments.

## miRNAs and drug resistance in cancer

Chemotherapy represents the primary treatment for both early and advanced tumors. However, drug resistance seriously limits the potency of conventional chemotherapeutics and novel biological agents, this constitutes a major obstacle in the treatment of cancer (1). Then, a lot of effort is aimed to identify new biomarkers, and to assess and predict the response of patients to drugs (2). Cancer drug resistance is referred as intrinsic, if tumors demonstrate to be insensitive to therapeutic agents before treatment, otherwise it is defined acquired if tumor becomes resistant during the treatment. The acquisition of resistance to several types of anticancer drugs can be due to the expression of transporters that eject drugs from cells, resulting in multidrug resistance (3). Nevertheless, several other mechanisms are involved in resistance, including insensitivity to apoptosis induced by drugs, increased repair of damaged DNA, decreased intracellular accumulation of therapeutics, and induction of mechanisms capable of drug detoxification (1). Recent data showed that other than by genetic and epigenetic changes, such as base mutations, amplifications, methylation and other post-translational modifications, drug resistance might also be due to non-coding RNA (ncRNAs) (4). The bulk of the human transcriptome, excluding the ribosomal and mitochondrial RNA, is represented by non-coding transcripts, including the most studied miRNAs and the newly discovered long non-coding RNAs (lncRNA) (5). MicroRNAs (miRNA) are small non-coding RNA molecules (18–22 nt in length) that act as negative regulators of gene expression through modulation of multiple target mRNAs, by inhibition of translation (6–9). A number of miRNA genes are located within intronic regions of genes, both coding or non-coding for proteins and can be transcriptionally regulated through their promoters (10). Other miRNAs are found either within exons, including 3′ UTRs of mRNAs, or clustered with other miRNA genes (11). Since their discovery (12, 13), the number of annotated miRNAs in the human genome has grown rapidly and they regulate a variety of cellular processes, including apoptosis (14), differentiation (15) and cell proliferation. miRNA deregulation has been demonstrated in cancer (16–19). The role of miRNAs in controlling cellular proliferation, differentiation and apoptosis, and their location at sites of translocation breakpoints or deletions (20), suggests that they might function as tumor suppressors or oncogenes (21–23). Profiles of miRNA expression differ between normal and tumor tissues, and among tumor types (18, 24–27). The association of miRNAs with cancer was first revealed in chronic lymphocytic leukemia (CLL), upon the discovery that miR-15a and miR-16-1 were frequently deleted or down-regulated (16, 28), and that their expression was inversely correlated to that of BCL2 (29). Since then, numerous studies have provided evidence for changes in microRNA expression in oncogenesis: different cancer pathways can converge to affect the same miRNAs and conversely a single miRNA can control an entire transcriptional program, affecting a lot of target genes. The deregulation of miRNAs is linked to cancer progression and clinical outcome (30), and miRNAs have been proposed as potential diagnostic markers, prognostics factors, and therapeutic targets (27, 31–33). When aberrant microRNA expression is directly involved in carcinogenesis (21), the inhibition of selectd microRNAs may have therapeutic implications. Modified antisense oligonucleotides have been designed *ad-hoc* and have proven effective at inhibiting microRNA function *in vivo* in mice (34, 35). The association of microRNA expression with cancer prognosis, therapeutic outcome and response to therapy, independently of other clinical covariates has been documented (25, 26, 36, 37), and selected miRNAs may influence cancer response to chemotherapy (38). The prognostic potential of microRNAs has been demonstrated for CLL (37), lung cancer (39), pancreatic cancer (25), and neuroblastoma (40) among others. One of the firsts observation on a possible link between miRNAs and drug resistance was reported in breast cancer (BC) suggesting that increased sensitivity of patients to anthracycline-based chemotherapy was related to deletion of chromosome 11q, a region containing MIR125B1 (41). The effect of miRNAs on chemotherapy was systematically studied by Blower et al. (42) on NCI-60, a panel of 60 cancer cell lines, used by the National Cancer Institute to screen >100,000 chemical compounds for anticancer drug sensitivity (20, 38, 42). Overall, miRNAs can mediate drug resistance through multiple pathways, including: (i) cell cycle and proliferation control, (ii) survival and/or apoptosis signaling pathways, (iii) DNA repair systems, (iv) specific drug targets, (v) adenosine triphosphate–binding cassette (ABC) transporter proteins, and/or drug metabolism, (vi) the epithelial–mesenchymal transition (EMT) process (4, 6, 43, 44). For example, miR-15b, miR-16 and miR-22 have been documented as mechanisms in chemotherapy resistance (45, 46). Cell cycle deregulation by miRNAs can induce resistance in cancer cells, as confirmed for miR-224 (47). Also, miR-24 and miR-508-5p can directly target enzymes involved in drug metabolism (48, 49). In addition to the mechanisms described above, modulation of epithelial-mesenchymal transition (EMT) can exert an effect on cancer cell resistance. Importantly, once cancer cells undergo EMT, chemo-resistance is increased and metastasis can occur (50, 51). Normal stem cells are already more resistant to drug treatment due to over-expression of drug efflux pumps and anti-apoptotic proteins (52). In this context, miR-34, miR-125b, miR-140, and miR-215 have an important role in conveying drug resistance to cancer stem cells (2). Chemotherapy can induce EMT and modulate the expression of miR-448 to promote cancer cell progression (53); conversely miR-29c or miR-224 have recently been shown to regulate the EMT process (54). miRNome dysregulation in relation to chemotherapy has been described for the most common tumor types: breast, ovarian, lung, prostate, gastric and colon cancer, squamous and hepatocellular carcinoma (HCC), cholangiocarcinoma, neuroblastoma and various types of leukemia (55). Overall, these studies highlight the complexity of adaptive/selective mechanisms in the establishment of resistance to cancer therapies.

## lncRNAs and drug resistance in cancer

lncRNAs have been linked to cancer progression and metastasis (56), and recently intensive research has been devoted to the molecular dissection of their roles, as well as to their diagnostic and prognostic significance (57). lncRNAs are mRNA-like transcripts 200 nt to ~100 kb in length lacking significant open reading frames. lncRNAs can be transcribed by RNA polymerase II (RNA pol II), poly-adenylated and located within nuclear or cytosolic fractions (58). lncRNAs can be divided into different categories: if overlapping with any transcript on sense or anti-sense strand lncRNAs will be classified as (i) sense or (ii) antisense respectively. When its expression is initiated along with a neighboring transcript, sense or antisense, that is proximal, (iii) bidirectional. When deriving from an intronic region, (iv) intronic or (v) intergenic if placed between two genes (53). Generally, lncRNA expression levels appear to be lower than those of protein-coding genes (54), and lncRNAs might be preferentially expressed in specific tissues (59). As to their functions, lncRNAs can regulate the expression of genes in close proximity (cis-acting regulation) or can target distant transcriptional activators or repressors (trans-acting) (53, 60). Their mechanisms of action are still diverse, and have been associated with a spectrum of biological processes, for example, epigenetics, alternative splicing, nuclear import, structural components, precursors to small RNAs and regulators of mRNA decay (60–63). Thus lncRNAs can regulate cellular functions such as chromosome dosage compensation (64), imprinting (65), cell cycle progression (66) and differentiation (67). Aberrant regulation of lncRNAs is reported in a variety of diseases, including cancer (68–71). Accumulating reports of misregulated lncRNA expression across numerous cancer types suggest that also this class of ncRNA can act in oncogenesis and tumor-suppression (72). A number of useful databases providing molecular information on lncRNAs are available (73). Loss of imprinting and redirecting chromatin remodeling complexes (74), induction of metastasis (75), depletion of miRNAs as “molecular decoy” or “miRNA sponge” (76) and direct inactivation of tumor suppressor genes (77) have been referred to specific lncRNAs. Preliminary studies commenced to report the value of ncRNAs as potential biomarkers in clinical settings (78, 79) and their roles in drug resistance (80).

## A network analysis: the most central ncRNAs in chemoresistance

**Figure 1:**
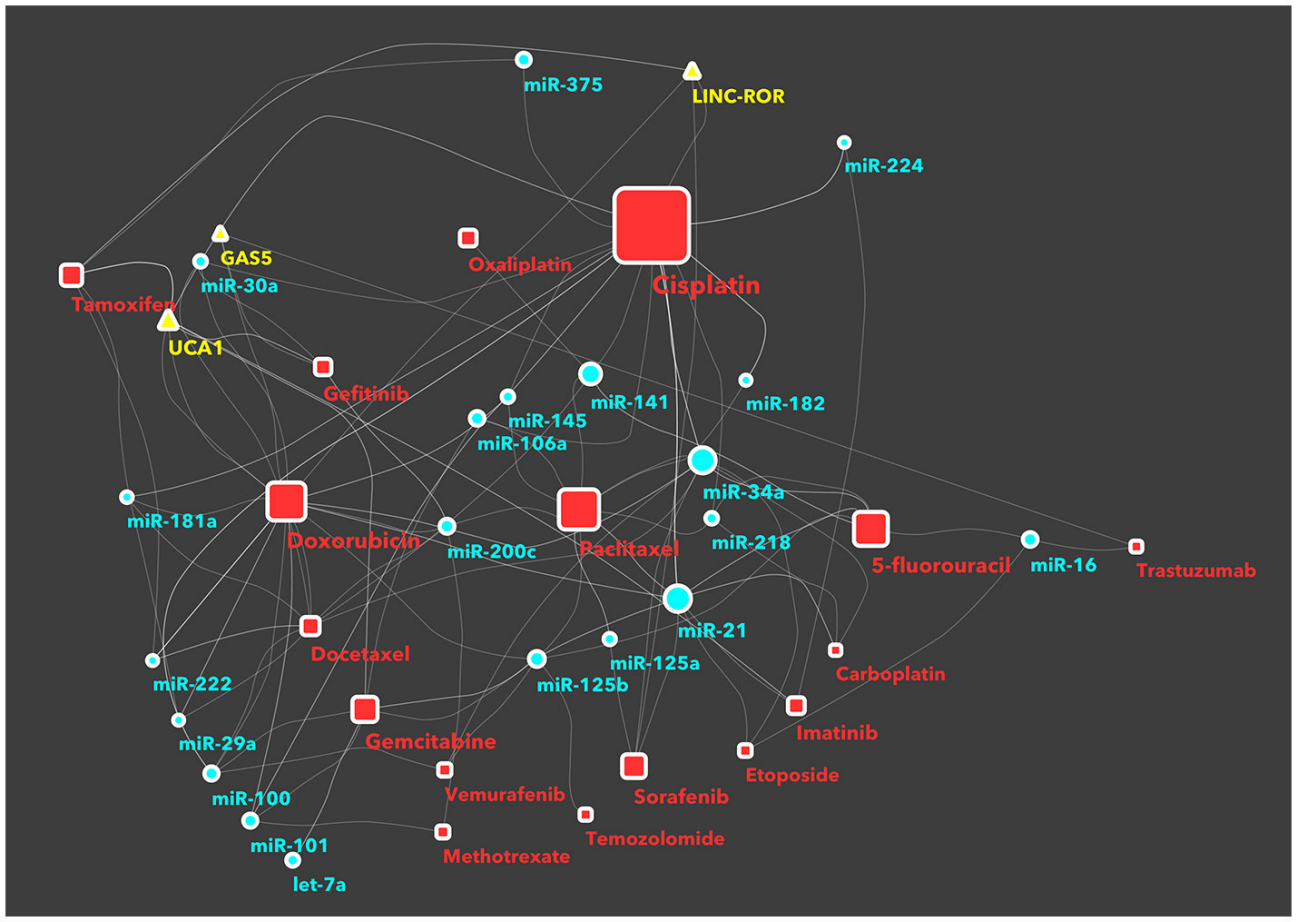
The network of non-coding RNAs and anti-cancer drugs. Each link between a drug and an ncRNA indicates a study in literature, investigating on the specific chemoresistance involvement of that ncRNA in cancer. The nodes (a ncRNA or a drug) shown in this figure have a degree >3, a central position in the network (expressed as betweenness centrality in the network description) >0.003, or a closer position relative to the companion drug (expressed as closeness centrality >0.26). The full network, with all nodes, is reported in supplemental information as Supplementary Image 1. Drugs are represented as red squares, miRNAs as light blue circles and lncRNAs as yellow triangles. The size of a node is proportional to its betweeenness centrality, while the size of a node name is proportional to its degree.

**Figure 2:**
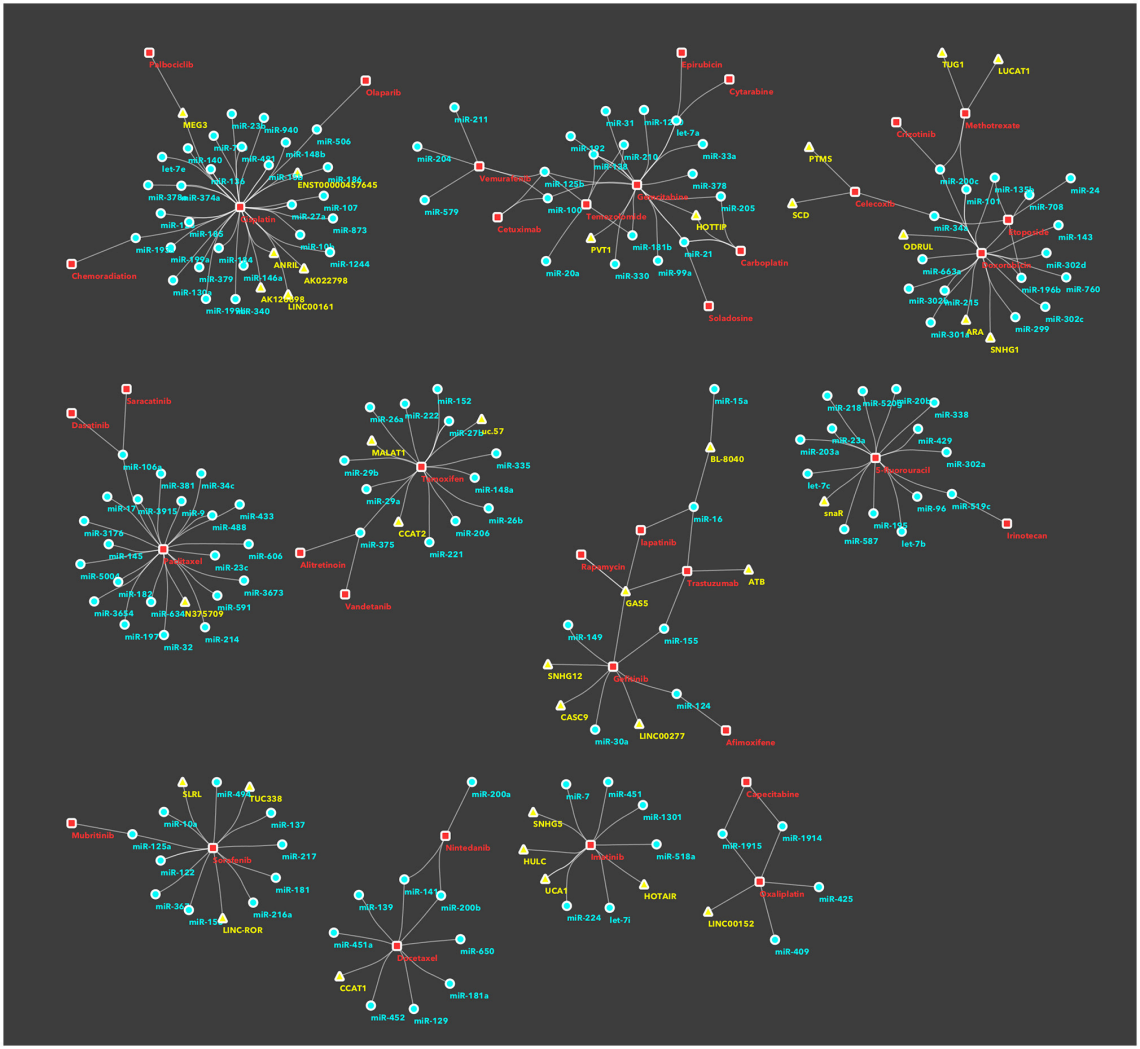
Subnetworks/clusters of non-coding RNAs/drugs associations, according to community analysis. This figure depicts disjoint subnetworks corresponding to the different clusters in the whole network (Supplementary Image 1) and identified using the community analysis tool in Cytoscape. This is a simplification of the involvement of ncRNAs in drug resistance, as an ncRNA, or a drug, is represented only in a single cluster/subnetwork. For sake of completeness, all interactions are described in the main text and presented in the Supplementary Table 1.

In recents years, an increasing number of studies have been reported on ncRNAs, target gene modulation, and affected drug functions, pharmacogenomics or chemoresistance. With the aim to facilitate the classification of ncRNAs and drug targets, some databases have been developed, such as NRDT (81) or Pharmaco-miR (82), collecting all information about ncRNA-target gene-drugs. There are large numbers, and growing, of both ncRNAs and cancer drugs, thus the combinations between members of the two groups are very difficult to manage in a traditional review or interpretate in a database. Therefore we decided to use machine-learning systems and to study the RNA-drug interactions using a network-based approach. Basically, we took from KEGG database all approved drugs used for cancer therapy. Then, we searched in PubMed all recent studies (published from 2011 onwards) investigating ncRNAs in chemoresistance. This selection was performed by batch analysis of PubMed-NCBI (National Center for Biotechnology Information) using as major topics the drugs from KEGG, ncRNA and chemoresistance. The result of this screening was manually curated in order to avoid and remove papers with generic statements and not direct links between ncRNAs and drugs. Only the investigations that proved (by *in vitro*/*in vivo*) experiments the existence of a direct association between ncRNAs and chemoresistance were then analyzed using a machine-learning tool. We thus built a network of non-coding RNAs starting from a human-curated selection of papers and applied an *ad-hoc* data mining approach to dissect the network and identify the most important ncRNA/cancer drugs interactions and cliques. We obtained a fully connected network of 388 drug/ncRNA interactions (edges) and 5 unconnected pairs (Supplementary Image 1). We then went on with studying the network, which had 227 nodes: 150 miRNAs, 35 lncRNAs and 42 drugs. Three graph theory measures were considered to define the most relevant non-coding RNAs associated to therapeutics resistance: (i) degree, indicating the number of links that an ncRNA had with different nodes (here drugs) (ii) betweenness centrality, a measure of centrality in the network based on shortest paths (iii) closeness centrality, related to the distance between the ncRNA and all the other nodes in the network. Then, we ranked the nodes (drugs and ncRNAs) and edges (combinations) in the network and collected the combinations from ncRNAs with a degree >3 and a central position (closeness centrality > 0.26 and betweenness centrality >0.003) (Figure 1 and Supplementary Table 1). Finally, we performed a community structure analysis using Glay and Cytoscape (83) to identify different clusters of ncRNAs and drugs. The clusters were converted to subnetworks for convenient visualization. The visual separation of clusters was improved by overlaying the community structure on a graphic layout addressing specific topology (Figure 2).

We wish to add a cautionary note to our reviewing effort. Even in the genome-wide studies (a minority among those we included in this review) for a number of conscious or unconscious reasons, scientists often end up chasing the most “popular” ncRNAs among others of “lesser pedigree.” Thus there is potentially a positive bias toward well-known ncRNAs in the overall scheme, and therefore in the final network. For this reason, we decided to keep all associations and, although the “degree” (number of associated drugs for an ncRNA) is important, we tried to avoid biased selections and included in our review all ncRNAs/pairs.

Here we commence with describing the ncRNAs that are most prominent in relation to chemoresistance, as detailed in (Figure 1).

**miR-21** has the highest scores (degree, betweenness centrality and closeness centrality) as it was associated with several drugs. The MIR21 gene is located at17q23.2, a region frequently amplified in several tumors (84, 85). Its overexpression has been observed in most cancer types and modulates the resistance toward apoptosis-inducing drugs (86–91). Down-regulation of miR-21 sensitizes cancer cells *in vitro* to different chemotherapeutics, including cisplatin, etoposide and doxorubicin (92–94). On the other hand, some drugs can induce alterations in miR-21 levels: e.g. soladosine can inhibit lung cancer cell invasion through miR-21 down-regulation, via PI3K/Akt signaling pathway (95). Interestingly, exogenous Epstein Barr virus modulates the PI3K/Akt pathway through LMP1, thus enhancing miR-21 expression and contributing to cisplatin reduced response in nasopharyngeal carcinoma (96). Moreover, miR-21 delivered by exosomes augmented malignancy in recipient cells and conferred paclitaxel resistance to ovarian cancer cells (97). There was also a report for enhancement of anticancer activity when Cao et al. reported that miR-21 induction sensitized gastrointestinal tumor cells to imatinib (98).

**miR-34a** was reported to be downstream of p53 and to function as a tumor suppressor (99). It is down-modulated in colorectal cancer (CRC) (100). In 5-Fluorouracil (5-FU)-resistant colon cancer cells ectopic expression of miR-34a inhibited cell growth and attenuated the resistance to 5-FU through down-regulation of SIRT1 and E2F3 (101), inhibition of LDHA (102) and of c-Kit, thus reducing stem cell factor (SCF)-induced migration/invasion (103). Yang et al. demonstrated that miR-34 targets BCL2 and sensitizes HCC cells to sorafenib (104). In osteosarcoma cell lines miR-34a has been tested in combination with celecoxib: the treatment showed decreased cell viability, migration and invasion through regulation of the Notch1/ROCK1-PTEN-Akt-GSK-3β axis (105). Moreover, miR-34 could enhance the therapeutic efficacy of paclitaxel in resistant prostate cancer (106). Its overexpression enhanced cisplatin sensitivity, as confirmed in gastric cancer, by targeting MET (107) and in lung cancer, through the p53/MICN axis (108). Conversely, Pu et al. found that miR-34a overexpression in osteosarcoma promoted resistance to several drugs (doxorubicin, etoposide, carboplatin, cisplatin), via repression of AGTR1 (109).

The **lncRNA Urothelial Cancer-Associated 1 UCA1** gene is located at 19p13.12 (110). Different transcriptional isoforms have been reported, UCA1 (1.4 kb), UCA1a (2.2 kb) and CUDR (2.7 kb), generated by alternative splicing and poly-adenylated. UCA1 is the most abundant isoform in various malignant tumors such as bladder cancer, BC and HCC (110–113). UCA1 could promote drug resistance by directly binding to miR-204, miR-18a and miR-16 (114). UCA1 emerged as a competitive endogenous RNA (ceRNA) of multi-drug resistance associated protein 1 (MDR1), inducing resistance to imatinib in CLL cells by sequestering miR-16 (115). Overexpression of UCA1 up-regulated MDR1, resulting in imatinib resistance, whereas its silencing had the opposite effect (116). In bladder cancer, UCA1 enhanced chemoresistance to cisplatin by regulating Wnt signaling (117) and to cisplatin/gemcitabine through modulation of miR-195a (118). Recent studies reported that UCA1 regulates tamoxifen resistance in BC (119). Liu et al. demonstrated that the knockdown of this lncRNA could revert resistant phenotype and increase tamoxifen sensitivity through inhibition of the Wnt/β-Catenin pathway, thus further confirming the oncogenic role of UCA1 in BC (120). Moreover, UCA1 was shown to be released in exosomes by tamoxifen resistant BC cells and increased tamoxifen resistance in ER-positive recipient cells (121).

The members of **miR-125** family (miR-125a, miR-125b-1 and miR-125b-2) play an important role in tumorigenesis and are potential biomarkers for cancer diagnosis, treatment and prognosis in clinical settings (122). MIR125A gene is on chromosome 19, while two separate loci on chromosomes 11 and 21 harbor MIR125B1 and MIR125B2, respectively (123). miR-125b expression has been found negatively correlated with 5-Fluorouracil resistance in HCC (124), while resistance to pharmacological treatments with gentamicin, cetuximab, doxorubicin and temozolomide by miR-125b still remains controversial (88, 125–127). miR-125b regulates the resistance to paclitaxel in colon cancer cells, in association with miR-125a (128). Recent data strongly supports a relevant role for miR-125b in conferring taxol resistance in BC, via suppression of pro-apoptotic BCL2 antagonist killer 1 (Bak1) (129). In contrast, in chondrosarcoma, overexpression of miR-125 enhanced the sensitivity to doxorubicin by directly targeting ERBB2-mediated glucose metabolism (130). miR-125a overexpression increased the response to paclitaxel in cervical cancer, through STAT3 down-modulation (131). Sorafenib treatment in HCC showed restoration of mir125 levels by sirtuin-7 and p21/p27 signaling blockage inhibiting cell cycle progression (132). In AML cells, via mubritinib, miR125a inhibited the ERBB pathway and cell cycle proliferation and progression, suggesting that miR-125a increased the sensitivity to the drug (133).

The MIR100 gene is at 11q24. Deregulation of **miR-100** has been reported in drug resistance; however, miR-100 expression can be either over-expressed or under-expressed in diverse cancers (134). In ovarian cancer, miR-100 targets mTOR therefore reverting the cell's chemoresistance toward cisplatin (135) and chondrosarcoma (136). In pancreatic cancer, miR-100 mimics inhibit proliferation and increase sensitivity to cisplatin by targeting FGFR3 (137). Recently, it has been shown that down-modulation of miR-100 could increase β-tubulin class V expression, promoting tumor cells proliferation, with implications for paclitaxel resistance (138). Also, miR-100 reduced ATM levels in a human glioma cell line (M059J) and could sensitize tumor cells to ionizing radiation (139). *In vitro*, miR-100 also induced the differentiation of BC stem cells expressing a functional ER (140). Furthermore, in CRC cells miR-100, together with miR-125b, negatively regulated Wnt/β-catenin signaling, and restored responsiveness to cetuximab (125). On the other hand, in mutant p53 pancreatic carcinoma, miR-100 up-regulation was related to gemcitabine resistance (88). In accordance, the exosomes-mediated intercellular transfer of miR-100, from drug resistant BC cells, could lead to resistance in sensitive cells (141).

**miR-200c** acts as a tumor suppressor, and could inhibit the initiating steps of metastasis; a negative correlation with *ZEB* factors has been reported, suggesting that this miRNA-mediated regulatory pathway influences EMT (142–147), potentially modulating drug resistance in advanced tumors. miR-200c reverses resistance of lung cancer cells, both to chemotherapeutics, like methotrexate (148), and to targeted drugs, like crizotinib (149) and gefitinib (146, 150). In breast and renal cancers, miR-200c could be involved in resistance or re-sensitization to microtubule-targeting drug (151–153).

**miR-141** is another member of the miR-200 family, also involved in EMT, invasion, migration and drug resistance (154). miR-141 overexpression contributes to acquired chemoresistance, for both *in vitro* and *in vivo* models. The initiation factor 4E (EIF4E) mRNA is a target of miR-141, that is involved in drug-induced apoptosis, conferring resistance to docetaxel-sensitive BC cells (155). miR-141 regulates cisplatin sensitivity in non-small lung cancer cells via PDCP4 inhibition and its inhibition increases cisplatin-induced apoptosis (156). In oesophageal squamous cell carcinoma, miR-141 was highly overexpressed in 5-Fluorouracil and oxaliplatin resistant cells and contributed to acquired chemo-resistance via PTEN (157). Moreover, in HCC cells, miR-141 was shown to confer resistance to 5-Fluorouracil through the inhibition of KEAP1, thereby reactivating the NRF2-dependent antioxidant pathway (158). Li et al. discovered that miR-141 together with other miRNAs like miR-16 contribute to prostate cancer chemoresistance via an exosome network (159).

Two homologous microRNAs, **miR-221** and **miR-222**, are generally considered having an oncogenic activity (160). The expression of miR-221 and miR-222 is highly up-regulated in HER2/neu-positive human BCs resistant to endocrine therapy, compared with HER2/neu-negative tissue samples (161); also, in BC patients miR-222 is elevated in chemoresistant tissues after surgery, compared with the pre-neoadjuvant samples (162). miR-221/222 reduce the protein level of the cell cycle inhibitor p27Kip1, conferring tamoxifen (161) and doxorubicin resistance (162). Also, secreted miR-221/222 could serve as signaling molecules and mediate communication of tamoxifen resistance (163). Aberrant expression of miR-222 is tightly related to poor overall survival (164) and affect oncogenic signaling pathways associated with resistance to different drugs (165). miR-222 also mediated BC cells resistance to adriamycin via PTEN/Akt/FOXO1 (164). Furthermore, the exosome mediated release of miR-222, miR-100 and miR-30a contributes to the same effect on docetaxel and doxorubicin: loss of responsiveness in BC cells (141). In oesophageal and prostate cancers, miR-221 could modulate 5-Fluoruracil resistance via the Wnt/β-catenin-EMT pathway (166) or RB1 (167), respectively.

**miR-101** (168, 169) has a relevant role in autophagy. Targeting the autophagy process is a promising therapeutic strategy to improve chemotherapy efficiency. In BC cells miR-101 inhibits basal autophagy, as well as etoposide- and rapamycin-induced autophagy, thus sensitizing cancer cells to 4-hydroxytamoxifen (4-OHT)-mediated cell death (170). In HCC, miR-101 sensitizes cell lines to cisplatin-induced apoptosis by targeting Mcl-1 (171). Likewise, miR-101 inhibits autophagy and enhances chemo-sensitivity to doxorubicin of osteosarcoma cells *in vitro* (172). In pancreatic cancer, miR-101 up-regulation reverts gemcitabine resistance by inhibiting the expression of ribonucleotide reductase M1 (RRM1) (173). Moreover, recent studies demonstrate that miR-101 interacts with lncRNA MALAT1 in regulatory networks that modulate cisplatin and temozolomide resistance, in lung cancer (174) and glioblastoma (80), respectively.

The **miR-15/16** gene cluster in chromosome 13 (13q14) is deleted or down-regulated in some cancer types (21). This somatic alteration was reported to occur early in cancer development and could represent a target for intervention (21). miR-16 expression is affected by several drugs: in gastric cancer cell lines etoposide and 5-Fluorouracil could increase the levels of miR-16, both *in vitro* and *in vivo*, and the up-regulation of miR-16 is modulated by p38 MAPK signaling pathway (175). In BC, lapatinib and trastuzumab are reported to regulate miR-16 via PI3K/Akt (176). Noteworthy, the altered expression of both miR-15a/16-1, due to the CXCR4 inhibitor BL-8040 induced the apoptosis of AML blasts by down-regulating ERK, BCL2, MCL1 and cyclin-D1 (177).

The lncRNA **GAS5**, originating from the Growth Arrest-Specific 5 gene, is down-regulated in multiple cancers. GAS5 inhibits proliferation and promotes apoptosis, thus playing a tumor suppressor role (178). Several studies confirmed GAS5 as an mTOR effector, and its expression was directly correlated with chemoresistance. Thus, enhancing GAS5 expression may improve the effectiveness of rapalogues, as confirmed both in prostate tumor cells and in mantle cell lymphoma cells (179, 180); also, the down-modulation of GAS5 caused resistance to trastuzumab in BC (181). In lung adenocarcinoma cells resistant to EGFR inhibitors, GAS5 enhance gefitinib-induced cell death, via down-regulation of IGF1R (182). Lastly, in bladder transitional cell carcinoma GAS5 inhibited malignant proliferation and chemotherapy resistance to doxorubicin, partly acting via BCL2 (183).

**miR-106a**, a member of the miR-17 family, is associated with poor prognosis, invasion and metastasis (184). In ovarian cancer (OV), miR-106a inhibited cell survival and cisplatin resistance, through downregulation of MCL1 (185); conversely expression of miR-106a was higher in cisplatin-resistant OV. miR-106a may be involved in the modulation of cisplatin-induced apoptosis by regulating PDCD4 (186). In non-small cell lung cancer, miR-106a also confers cisplatin resistance, by targeting adenosine triphosphatase-binding cassette A1, an ABC transporter (187). Otherwise, by targeting autophagy, miR-106a enhances sensitivity of lung cancer cells to SRC inhibitors, including saracatinib and dasatinib, expliciting once more the context-dependent function of miRNAs (188). Further, dysregulation of miR-106a conferred resistance to paclitaxel in OV; its modulation resensitized resistant cells by targeting BCL10, caspase-7, and ZEB1 (189). Down-modulation of miR-106a was reported in gentamicin resistant hepatoma, participating to EMT via the PDGF-D/miR-106a/Twist1 pathway; notably, in HCC patients, miR-106a and Twist1 were associated with PDGF-D expression (190).

**miR-375** is involved in a positive feedback loop with ER in BC (191) and its re-expression is sufficient to sensitize tamoxifen-resistant cells. Furthermore, miR-375 partly reversed the EMT process: metadherin (MTDH) was identified as a direct target of miR-375 and tamoxifen-treated patients with higher MTDH had a higher risk of relapse (192). Another miR-375 target is HOXB3; miR-375 inhibited cancer stem cells (CSCs) phenotype and tamoxifen resistance by regulating CSCs, through degradation of HOXB3 (193). Epigenetically down-regulated miR-375 in HER2-positive BC could induce trastuzumab resistance by targeting IGF1R (194). 9-cis retinoic acid (Alitretinoin) modulated the expression of miR-375 in BC depending on ER status: thus, miR-375 was inhibited in ERα-positive cells while highly induced in ERα-negative cells (195). The deregulation of miR-375 was also observed in other malignancies: in medullary thyroid carcinomas (MTC) miR-375 was the strongest up-regulated miRNA (196). Vandetanib is a tyrosine kinase inhibitor for the treatment of patients with recurrent or metastatic MTC that are unresectable, and/or symptomatic (197). Interestingly, miR-375 over-expression associated with SEC23A down-regulation could improve the efficacy of vandetanib (196). Thus, the expression levels of miR-375 and SEC23A pointed to vandetanib sensitivity and could be evaluated as predictive indicators for efficacy of vandetanib in MTC. Analogously, up-regulation of miR-375 increased the cisplatin-sensitivity of gastric cancer cells by regulating ERBB2 and phospho-Akt (198).

A role in chemoresistance modulation has emerged for putative tumor-suppressor **miR-145** (199). miR-145 targeting of MDR1 helps to restore drug efficacy in resistant cells and *in vivo* models of bladder cancer and BC (200, 201). Moreover miR-145 confirmed its role in reducing chemoresistance also with paclitaxel (202) and doxorubicin (203), possibly via regulation of EMT.

**miR-218** has a physiological role in neuron development and its loss of expression is involved in neurodegeneration (204). In BC, it acts as a risk factor in ductal carcinoma *in situ* (DCIS) (205). In association with platinum compounds, miR-218 and miR-205 inhibit tumorigenesis and overcome chemoresistance in lung cancer (206). In prostate cancer, miR-218 up-regulation inhibited tumor growth and increased chemo-sensitivity to cisplatin, by negatively regulating BCAT1 (207). Furthermore miR-218 mediated autophagy and was associated with positive response to paclitaxel in resistant endometrial carcinoma (208). It also promoted apoptosis and caused cell cycle arrest in CRC by targeting BIRC5, thus possibly enhancing first-line 5-FU treatment. Also, miR-218 through targeting the enzyme thymidylate synthase (TS), enhanced 5-FU cytotoxicity in CRC cells (209).

The **let-7** family members are down-regulated in lung (210), gastric (211), colon cancer (212) and in Burkitt's lymphoma (213). Loss of let-7 was associated with the shortened post-operative survival of patients with lung cancer (210). The altered expression of let-7a could increase chemoresistance to epirubicin (214) and cytarabine (215). Furthermore, let-7a expression has demonstrated to influence chemoresistance, due to maintained treatment with gemcitabine, in pancreatic cancer patients (216, 217). Several studies have reported that let-7a acts as a tumor suppressor in renal cell carcinoma (RCC), by targeting c-Myc (218). let-7b and let-7e are down-regulated in glioblastoma and ovarian cancer, respectively and promote resistance to cisplatin by acting on the same target Cyclin D1 (219, 220). Reduced levels of both let-7b and let-7c could determine the intrinsic chemoresistance to 5-FU in RCC, possibly via AKT2 (221). Clinically, 5-FU-based chemotherapy is considered moderately effective in RCC due to rare response and severe toxicity (222); transfection of let-7b or let-7c potentiated the efficacy of 5-FU *in vitro* at tolerable concentrations. Moreover, let-7c up-regulation contributed to sensitize lung cancer cells with acquired cisplatin resistance, by involving ABCC2 and Bcl-XL (223). Interestingly, a combination of **miR-224 and let-7i**, reduced imatinib resistance in CML, probably through targeting the ST3GAL IV sialyltransferase (224).

**miR-30a** was found to act as an oncosuppressor, but could also promote tumor progression in several types of cancer (225). The same dual activity was described for drug resistance. In ovarian and lung carcinoma miR-30a interacted with cellular receptors (EDNRA and EGFR) and played an important role in overcoming the acquired resistance (226, 227), also via exosomes (141). **miR-181a** is down-regulated in glioma and lung cancer, while its up-regulation is involved in metastasis and invasion in breast and oral squamous carcinomas (228). Prostate cancer patients undergoing maintained treatment with taxane develop resistance to the therapy. Recently, Armstrong et al. discovered that miR-181a overexpression contributes to docetaxel and cabazitaxel resistance in prostate cancer cells (229). The role of miR-181a in cisplatin resistance is apparently dual: in cervical squamous cancer, it could induce chemoresistance, partly by down-regulating PRKCD (230), while it could reverse cisplatin resistance in tongue squamous cell carcinoma, acting through Twist1 (231).

**miR-182** is overexpressed in a broad range of tumor types. Clinical studies associated miR-182 with increased aggressiveness and poor survival (232). miR-182 was also found to have a role in chemoresistance. Acting as a negative regulator of PDCD4, it determined a reduction of sensitivity to cisplatin and paclitaxel in OV (233) and to cisplatin in lung cancer (234). Further, in HCC miR-182 was directly correlated *in vitro* and *in vivo* with cisplatin resistance, possibly by regulating TP53 (235).

In inflammatory bowel disease (IBD) and in cancer, **miR-224** has an important function. By targeting p21, it participated in cell cycle regulation at the G1/S checkpoint (236). miR-224 could induce resistance to cisplatin in lung and ovarian cancer cell lines (47, 237). In contrast, miR-224 promoted cisplatin sensitivity in osteosarcoma resistant cells by targeting Rac1 (238). miR-224 was related with CRC progression and the response to 5-fluorouracil through KRAS-dependent and -independent mechanisms (239).

Finally, **miR-29** family members are miRNAs that can play different roles in cancer (240). For example, they can contribute in BC to the acquisition of doxorubicin resistance by inhibition of PTEN/AKT/GSK3β (241). Conversely, miR-29b exerts a tumor suppressor activity in tamoxifen-resistant BC cells (242).

The lncRNA Regulator Of Reprogramming **LINC-ROR** is involved in the regulation of the pluripotent stem cells reprogramming. Its expression suppresses the induction of p53 after DNA damage and is associated with tumor progression, EMT and metastasis (243). LINC-ROR is significantly up-regulated in BC, resulting in chemotherapy tolerance and enhanced invasiveness (244). In tamoxifen-resistant BC cell lines, down-regulated LINC-ROR could inhibit EMT and enhance the sensitivity to tamoxifen by increasing miR-205 (245). A relevant study on cancer tissues from BC patients demonstrated that inhibition of LINC-ROR reversed resistance to tamoxifen by inducing autophagy (246). Moreover, LINC-ROR could mediate for sorafenib chemosensitivity in HCC, through the realease of extracellular vesicles (247).

## Drugs/non-coding RNAs subnetworks

Non-coding RNAs can regulate several protein targets or molecular pathways that lead or inhibit drug resistance according to tumor type, stage and class of drug (248). Above we discussed the ncRNAs with the most prominent roles in the literature as measured using network statistics. There are though many ncRNAs which have been described only in association to one or few more drugs: for these rare ncRNA/drug combinations we performed a clustering analysis of the whole network and identified less than a dozen of groups. The ncRNA/drug combinations are described below as subnetworks and are visualized in (Figure 2). The Supplementary Table 2 details the effects of ncRNAs on chemoresistance.

### Subnetwork 1: gefitinib, afimoxifene, rapamycin, trastuzumab, lapatinib, BL-8040

Gefitinib is a selective inhibitor of the Epidermal Growth Factor (EGFR) protein. It is used to treat solid tumors, as non small cell lung cancer (NSCLC). It acts by inhibiting the anti-apoptotic Ras signaling cascade (249). Recent studies confirmed also that the loss of regulation of ncRNAs is involved in chemoresistant acquisition (250, 251). The GAS5 lncRNA is implicated in chemoresistance modulation of several different drugs included into this subnetwork (179–182). Another interesting lncRNA present in this group is the Small Nucleolar RNA Host Gene 12 (SNHG12), that plays an oncogenic role in various cancers (252). Moreover, SNHG12 overexpression is implicated in multidrug resistance (included gefitinib resistance), by sponging miR-181a and thus activating the MAPK/Slug pathway (253). This confirms also the involvement of miR-181a in the regulation of chemoresistance. miR-16 has been previously described, and in cancers it may regulate the response to trastuzumab and lapatinib. This miRNA plays an important role in inhibiting cell proliferation and potentiting drug effects (176). Furthermore, in leukemia miR-16 in combination with miR-15 interacts with new phase II drug (177). miR-124 has a role in neuronal differentiation (254) and may modulate resistance to gefitinib and afimoxifene: miR-124 down-regulation could reverse afimoxifene induced autophagy in BC through regulation of Beclin-1 protein (255), while in lung cancer miR-124 depletion plays a role in gefitinib resistance by regulating SNAI2 and STAT3 expressions (256). A prolonged treatment with Gefitinib dramatically reduced the expression of miR-155 and miR-200c. The depletion of these miRNAs may contribute to the decrease in the sensitivity to gefitinib (150). Intriguingly, trastuzumab positively regulates miR-155 and as a consequence, this micro RNA negatively regulates ErbB2 and the malignant cell transformation of breast epithelial cells (257).

### Subnetwork 2: cisplatin, olaparib, palbociclib, chemoradiation

Cisplatin is a platinum compound classified as alkylating like agent that interferes with DNA replication and is used to treat several solid malignancies (258). The efficacy of cisplatin in cancer therapies is limited by the acquired resistance, that can lead to therapeutic failure and tumor recurrence (259). It was demonstrated that cisplatin-resistant cancer cells present an altered expression pattern of ncRNAs (260–281). Among them, miR-451 is known to exert a critical role in the pathogenesis and the development of several types of cancers, including CRC, glioblastoma and NSCLC. miR-451 is located on chromosome 17q11.2, in close proximity of ERBB2 (17q12) (282). miR-451 enhances cisplatin sensitivity in lung cancer cells through regulation of Mcl-1 (283); furthermore, it is involved in the resistance to imatinib in CML patients (284). Another ncRNA present in this network is miR-20a, a member of miR-17 family, which has an oncogenic role and is involved in leukemia and CRC (285). Moreover, Zhou et al. established that miR-20a expression in glioma cells was negatively correlated to Temozolomide sensitivity by targeting DNA methyltransferase (DNMT1) (286). In gastric cancer, miR-20a negatively regulates cylindromatosis (CYLD) expression, thus inducing cisplatin resistance (287). miR-15b had a dual role in oral tongue squamous cell carcinoma (TSCC) and lung adenocarcinoma; through the regulation of TRIM14 it was implicated in the reversion of cisplatin resistance in TSCC (288), while it decreased sensitivity to cisplatin by targeting PEBP4 in lung adenocarcinoma (289). Similarly, Chen et al. discovered the involvement of miR-136 as a tumor suppressor, which targeted E2F1 gene and reversed cisplatin resistance in glioma cells (290). On the contrary, in ovarian cancer miR-136 might induce chemoresistance through the inhibition of apoptosis, while promoting the repair of cisplatin-induced DNA damage (291). miR-27 has a well-defined role: in lung adenocarcinoma cells it contributed to cisplatin resistance by suppressing Raf Kinase Inhibitory Protein RKIP (292). Strikingly, in esophageal cancer miR-27 was associated with the transformation of normal fibroblasts to cancer-associated fibroblasts (293). The same ncRNA could have a role in the sensibilization to different drugs: e.g., miR-506-3p, which is up-regulated in ovarian cancer, has an important function in sensitizing cancer cells to both olaparib and cisplatin (294). Another interesting example is miR-193a-3p that can contribute to the inhibition of chemoradiation and of cisplatin resistance through PSEN1 and p73, respectively in esophageal tumor (295) and osteosarcoma (296). These findings confirmed an oncosuppressor activity for miR-193a-3p (297). miR-199 also may act as either a potential tumor suppressor or oncogene depending on cellular context (298). Consequently, epigenetic silencing of miR-199b-5p may contribute to raise cisplatin resistance via loss of control in cell cycle regulation (299) or miR-199a-3p may enhance cisplatin sensitization by downregulating TFAM (300). Interesting situations emerged when comparing miRNAs from the same family: i.e. down regulation of both let-7 members (let-7b and let-7e) controlled cisplatin resistance through down-modulation of cyclin D1 (219, 220). lncRNAs are an eterogeneous class of non coding RNAs and several studies demonstrated that their dysregulation could affect chemoresistance modulation as much as miRNAs (301–303). Maternally expressed 3 (MEG3) lncRNA that acts as a growth suppressor in tumor cells and selectively regulates p53 target (304), does not have a defined role in chemotherapy. Nevertheless, its up regulation seems to enhance cisplatin resistance in lung cancer (305). Meanwhile, palbociclib can determine the increment of MEG3 expression in a dose dependent manner, yielding to an increase anticancer outcome (306). Controversely, lncRNAs might also modify drug responsiveness exerting a miRNAs sponge activity acting as ceRNAs. Wang et al. demonstrated that downregulation of ANRIL lncRNA enhanced cisplatin citotoxicity via let-7a in nasopharyngeal carcinoma (307). These findings further confirm the role of let-7 family as inhibitors of chemoresistance.

### Subnetwork 3: paclitaxel, saracatinib, dasatinib

Subnetwork 3 incorporates several non-coding RNAs related with paclitaxel. This antineoplastic drug is a taxol derivative that blocks cell cycle progression by targeting beta-tubulin. Paclitaxel causes inhibition of mitosis and triggers the apoptotic process or the reversion of cell cycle. Paclitaxel is used to treat a number of solid cancers that include lung, ovarian, breast and pancreatic tumors (308). A number of studies produced evidence that loss of non-coding RNAs regulation can modify chemoresistance to taxol (202, 309–313). miR-182 is often up-regulated in cancers; it can enhance cell proliferation, invasion and it plays an important role in drug resistance. Two different studies found that miR-182 overexpression, by negatively regulating programmed cell death 4 (PDCD4), was involved in chemoresistance exacerbation of lung and ovarian cancers to cisplatin and paclitaxel, respectively (233, 234). Qin et al. demonstrated also that miR-182 expression increases cisplatin resistance of HCC cell by targeting TP53INP1 (tumor protein 53-induced nuclear protein 1) (235). miR-214, through targeting activating protein 2 (AP-2), contributes to regulate molecular processes in melanoma (314). Despite its role, miR-214 function in chemoresistance is still not clear: it could enhance sensitivity to cisplatin in esophageal cancer (315), or promote paclitaxel and carboplatin resistance in ovarian cancer (89). miR-9 may influence cell growth, cell cycle and it is often downregulated in cancer (316). miR-9 down-regulation is one of the key mechanisms accounting for paclitaxel resistance in ovarian carcinoma (317); while high expression of miR-9 in CD133+ glioblastoma cells activates MDR1 gene and imparts Temozolomide (TMZ) resistance (318). miR-17-5p isan oncogenic miRNA, member of the miR-17~92 cluster, which plays an important role in the control of cell cycle progression (319). Despite its oncogenic role, miR-17-5p can promote paclitaxel-induced apoptosis by increasing p53 expression in BC cells (320). The same ncRNA may also influence resistance to different drugs. It is the case of miR-106a that can enhance paclitaxel resistance through apoptosis inhibition (189) or promote sensitivity of lung cancer cells to Saracatinib and Dasatinib (188). In addition, the secretion of miRNA in exosomes is involved in paclitaxel resistance of prostate cancer (159).

### Subnetwork 4: sorafenib, mubritinib

To treat HCC in advanced status the multikinase inhibitor Sorafenib is the only validated therapy, but tumor response rates to this drug are quite low (321). Several miRNAs, including miR-137 (322), miR-367-3p (323), and miR-125a (131, 133) or lncRNA such as LINC-ROR (247) are involved in the regulation of HCC-Sorafenib treatment efficacy. Tang et al. demonstrated that the simultaneous silencing of miR-21, miR-153, miR-216a, miR-217, miR-494, and miR-10a-5p overcome sorafenib resistance *in vitro* and *in vivo* models of HCC (324). Azumi et al. found also that up-regulation of miR-181a increased sorafenib resistance, by blocking a MAPK signaling factor (RASSF1) in HCC cells (325). miR-122 is highly expressed in the liver, where it has been implicated as a regulator of fatty-acid metabolism. This ncRNA was significantly reduced in sorafenib-resistant HCC cells. Xu et al. demonstrated that miR-122 restoration increases sensitivity to sorafenib and induces apoptosis by repressing IGF1R (326). miR-122 is also involved in the control of arginine transport by targeting the solute carrier family 7 (SLC7). Arginine is the substrate for nitric oxide (NO) synthetase and as a result, loss of miR-122 in HCC cells causes an increment of intracellular NO and resistance to sorafenib (327). Moreover, knock-down of TUC338 lncRNA increased expression of RASAL1 protein in HCC, inhibited tumor growth and sensitized cells to sorafenib (328). Sorafenib is also used in the treatment of renal carcinoma (RCC), where SRLR (sorafenib resistance-associated lncRNA in RCC) was found up-regulated in sorafenib-resistant RCCs and contributed to sorafenib tolerance (329).

### Subnetwork 5: docetaxel, nintedanib

Docetaxel is a drug that promotes cell apoptosis after its interaction with beta-Tubulin metabolism and Bcl-2 phosphorylation. It is used to treat late-stage and metastatic BC, head and neck cancer, stomach cancer, prostate cancer and NSCLC (330). This subnetwork underlines the role of miR-129, a miRNA with tumor suppressor activity in several cancers (331). Lu et al. confirmed the role of this miRNA also in reducing drug resistance: miR-129 in gastric cancer cells reverses cisplatin-resistance through inhibition of P-gp expression (332). Nevertheless, another study demonstrated that miR-129 overexpression may be implicated in BC and docetaxel resistance, mainly through CP110 inhibition (333). Up-regulation of miR-141 and miR-181a (155, 229) also could contribute to docetaxel resistance, while down-regulation of miR-29a and miR-451 inhibited this process (334). Similarly to miR-200c, miR-200b has also a role in drug response: loss of miR-200b regulated autophagy in lung adenocarcinoma and was associated with resistance to docetaxel (335). Nintedanib inhibited VEGFR and consequently angiogenesis (336, 337). Nintedanib is also capable of reverting the resistance to gefitinib promoted by miR-200b and miR-141 (338). Dongqin et al. found that miR-451 down-regulation induced c-Myc expression, an event related to docetaxel-resistance (339). The role of miR-139 in cancer is still not clear (340), but by targeting NOTCH1, it could mediate cell sensitivity to docetaxel and 5-FU, respectively in breast (341) and CRC (342). Chen et. al. reported that miR-30a was related with docetaxel resistance in BC by horizontal exosomes transfer (141). Aberrant expression of CCAT1 lncRNA had a sponging effect on miR let-7c and, as a consequence, promoted chemoresistance to docetaxel in lung adenocarcinoma (343). This last evidence is intriguing, since it is also reported that let-7c up-regulation inhibited chemoresistance to 5-Fluorouracil in renal carcinoma (221) and sensitized resistant lung carcinoma cell (A549) to cisplatin (223).

### Subnetwork 6: gemcitabine, temozolomide, cetuximab, carboplatin, cytarabine, epirubicin, soladosine, vemurafenib

Gemcitabine is a synthetic nucleoside analog used to treat various carcinomas and several investigations confirm that ncRNAs can modulate gemcitabine action (344, 345). Cao et al. demonstrated that miR-192 regulated gemcitabine and cisplatin resistance in lung adenocarcinoma through modulation of apoptosis (346). miR-192 together with miR-215, was found to be a positive regulator of TP53 (347). In glioblastoma miR-138 is involved in cell death mechanisms that promote chemoresistance to temozolomide (348), an alkylating drug similar to gemcitabine, that enhances cell apoptosis of tumor cells. Moreover miR-138 aberrant expression can provide a basis for novel gemcitabine chemoresistance markers in bladder and pancreatic ductal carcinoma (88, 349). Furthermore, miR-138 was implicated in the pathogenesis of chronic myeloid leukemia and its clinical response to imatinib (350). Cetuximab is a monoclonal antibody with a mechanism of action different from gemcitabine, however, miR-100 over-expression may promote chemoresistance against both treatments (88, 125). Depending on the cellular context both up-regulation and down-regulation of an ncRNA could lead to chemoresistance. In our review, the role of miR-205, which regulates EMT (351), emerged as one of such cases. It is apparent that miR-205 upregulation causes inhibition of chemoresistance to gemcitabine in pancreatic cancer (352), but Zarogoulidis et al. demonstrated that miR-205 and miR-218 were associated with carboplatin resistance in lung cancer (206). miR-181b over-expression increased gemcitabine resistance (353), whereas miR-181b was involved in temozolomide sensitivity in glioma by targeting MEK1 (354). Lee et al. found that hypoxia-induced miR-210 (355) could potentially reverse temozolomide resistance in glioblastoma (356). Another investigation discovered that miR-210, in association with miR-21, miR-99a, miR-100, miR-125b, and miR-138 may serve as biomarkers of gemcitabine resistance in pancreatic cancer (88). In this subnetwork we find also some important miRNAs that were previous described: miR-21-5p (91) miR-125b-5p (89, 125, 127), and let-7a (214–217). The HOTTIP lncRNA (HOXA transcript at the distal tip) can promote cancer progression and gemcitabine resistance in pancreatic cancer (357). Finally the overexpression of BC200 lncRNA has a role in the induction of cell death by carboplatin in ovarian cancer (358). Furthemore, miR-204 is highly induced by vemurafenib in resistant melanoma cells and tissues, as much as miR-211 (359). Although belonging to the same family, the expression of miR-204 is high in amelanotic melanoma cells, and acts as an effector of vemurafenib's anti-motility activity. Conversely, miR-211 which is induced in melanotic melanoma cells, mediates and potentiates the increase in pigmentation due to vemurafenib; this adaptive response *de facto* limits its efficacy (360). miR-204 inhibits the migration/invasion of melanoma cells with a potency similar to that of miR-211 and, more importantly, it acts in the cellular contexts in which miR-211 is absent (359). Fattore et al. demonstrated that miR-579-3p is strongly downregulated in melanoma and loss of BRAF and MDM2 regulation leads to chemoresistance to targeted therapy (361).

### Subetwork 7: oxaliplatin, capecitabine

Oxaliplatin is used for the treatment of CRC and has been compared with other platinum compounds used for advanced cancers, such as cisplatin and carboplatin. Oxaliplatin in combination with capecitabine (XELOX) is a first-line treatment of CRC, hile for CRC in advanced stages is common to use oxaliplatin in combination with 5-FU (FOLFOX) (362). Several studies demonstrated that miRNAs modulate the chemoresistance to these drugs. In particular, Hu et al. found that circulating miR-1914-3p and miR-1915-3p are down-regulated in patients with chemoresistant CRC. Consequently, up-regulation of these miRNAs *in vivo*, could partially restore CRC cells sensitivity to XELOX treatment (363). Furthermore, miR-425-5p ihnibition reversed oxaliplatin resistance both in HTC116-resistant cells lines and xenograft models by modulating the expression of PDCD10 (364). Tan et al. observed a negative correlation between miR-409-3p and resistance to Oxaliplatin in CRC resistant cells (365). Moreover, as a putative miRNAs modulator, also long intergenic noncoding RNA (LINC00152), can be involved in chemosensitivity of Oxaliplatin in CRC. LINC00152 increases the chemosensitivity becoming an endogenous RNA competitor for miR-193a-3p and ErbB receptor tyrosine kinase 4 (ERBB4) (366).

### Subnetwork 8: 5-fluorouracil, irinotecan

5-Fluorouracil (5-FU) is a widely used therapeutic agent for treating a range of cancers, including advanced CRC (367), liver and BCs. It interferes with DNA replication by interrupting the synthesis of pyrimidine thymidine and thereby leading to cell cycle arrest or cell death (368, 369). In the 5-FU metabolic pathway, the enzymes dihydropyrimidine dehydrogenase, thymidylate synthase, thymidine phosphorylase and methylenetetrahydrofolate reductase are important to determine resistance (370). miRNAs are altered in CRC (26) and targeting tumor-associated genes (23, 371–373). Moreover, miRNAs are promising tumor biomarkers for CRC screening (27) and are also responsible for 5-fluorouracil drug resistance (374). In particular miR-587 (369), miR-195 (375), miR-149 (376), miR-203 (377), miR-129 (378), and miR-218 (209) are involved in 5-FU response. While miR-20b (379) and miR-519c (380) influence 5-FU and Irinotecan (only miR-519c) resistance in CRC. Another interesting miRNA is miR-302a, belonging to the miR-302-367 cluster, which includes miR-302b, miR-302c, miR-302a, miR-302d, and miR-367. This cluster was first identified in human embryonic stem cells (hESCs) and human embryonic carcinoma cells (hECCs) and it has been reported to help maintaining stemness and reprogramming somatic cells into induced pluripotent stem cells (381). Recently, *in vitro* models have pinpointed its role in chemoresistance: miR-302a exerts its function through inhibition of IGF1R and of downstream Akt signaling: events associated with enhanced 5-FU-induced cell death in colon cancer cells (370). The up-regulation of miR-96 has been reported in several cancers (382, 383) and conversely low expression levels of miR-96 have been associated with poor clinical outcomes in CRC patients (384). miR-96 modulated 5-FU sensitivity in CRC cells by promoting apoptosis through reduction of the anti-apoptotic regulator XIAP and the p53 stability regulator UBE2N (ubiquitin-conjugating enzyme E2N) (385). miR-23a antisense enhanced 5-fluorouracil chemosensitivity in CRC cells, by acting on the APAF1/Caspase-9 apoptotic pathway (386), while miR-23a over-expression provided 5-FU resistance in a subtype of CRC (387). Like let-7c, also present in this subnetwork, let-7b resulted important for development of 5-FU chemoresistance in RCC (221). miR-34a also plays a role in resistance to 5-FU and to vemurafenib (102, 103, 388). The expression profile of lncRNAs was investigated in 5-FU-resistant colon cancer cell lines and snaR was confirmed to be downregulated (389); this loss increases cell viability after 5-FU treatment, suggesting that this lncRNA has a potential role as a negative regulator in drug response (390). miR-204 is significantly attenuated in CRC (391) and has a relevant function in this cancer as tumor-suppressive miRNA, through direct targeting of HMGA2. The miR-204/HMGA2 axis notably modulated cell proliferation and positively influenced CRC sensitivity to 5-FU (392).

### Subnetwork 9: doxorubicin, methotrexate, etoposide, crizotinib, celecoxib

Most of non-coding RNAs disregulations related to doxorubicin, methotrexate and etoposide play a role in chemoresistance exacerbation or inhibition. They are involved in several pathways that regulate cell growth, autophagy, apoptosis and cell proliferation (393–401) or miR-34a (105), lnc-SCD and lnc-PTMS (402) modulates the effects of celecoxib. Both doxorubicin and etoposide block DNA replication by topoisomerase II inhibition: thus causing errors in DNA synthesis and promoting apoptosis in cancer cells. They are often used to treat cancers including breast, bladder, ovarian, prostate and leukemia (403). The human miR-135a is encoded by two genes localized on chromosomes 3 and 12. It may have contradictory effects promoting or repressing cell migration and invasion in colon, melanoma, breast and prostate cancer cell lines (404). This subnetwork shows a relation between miR-135 and miR-196b; upregulation of these two miRNAs is reflected in ABCB1 increment. This pattern conferred resistance to genotoxic agents like etoposide and doxorubicin in leukemia cancer cells (405), an interesting result that confirms the pro-oncogenic role of miR-196b. Its over-expression has been reported in different types of leukemia (406), in the maintenance of stem cell properties and chemoresistance in CRC (407), and in castrate-resistant prostate cancer (408). Novel insights in improving the effectiveness of chemotherapy emerged with miR-708, miR-101-3p, and miR-29b. Their regulation could enhance chemosensivity of drug targeted genes involved in responses like autophagy or apoptosis (172, 409, 410). miR-29b is generally the most highly expressed ncRNA in the miR-29 family. Up-regulation of miR-29b is common in the majority of human cancers where it affects tumor progression (411). miR-29b increases etoposide and paclitaxel induced toxicity in ovarian cancer, this effect being linked to Mcl-1 (410, 412). Very interesting was the case of the miR-200 family members that include miR-200c. The expression of this miRNA was inversely correlated with the chemoresistance to antioneoplastic drugs like Doxorubicin, Crizotinib and Methotrexate. miR-200c improved drug sensivity targeting TrkB and Bmi1 in BCs (151), ZEB1, and EZH2 in lung cancer cells (148, 149). Furthermore, Ham et al. found that overexpression of LUCAT1 lncRNA promotes methotrexate resistance through miR-200c (413). A very interesting loop, if considered that miR-200c up regulation contributes to restore methotrexate sensivity. The identification of ncRNA effects on cancer drugs could promote the development of novel approaches. For example, Xu et al. found that co-delivery of miR-101 and doxorubicin suppressed malignant properties of HCC (414). The role of miR-215, as well as that of his homologous miR-192 (subnetwork 6), in cancer is ambiguous. These two miRNAs exert cell growth and migration-promoting effects in gastric cancer (415) and are positive regulators of p53, playing an important role in multiple myeloma (347). Furthermore, a recent study has confirmed that miR-215 overexpression leads to the development of doxorubicin resistance in HCC and is also associated with bad prognosis in HCC patients harboring mutated p53 (416). In another case, Doxorubicin was shown to affect the subcellular localization of lncRNAs and to enhance their functional effects. For example, Shen et al. discovered that SNGH1 was retained in the nucleus as a consequence to doxorubicin treatment, in turn leading to accumulation of p53 in the nucleus and to the enhancement of p53-dependent apoptosis (417).

### Subnetwork 10: tamoxifen, vandetanib, alitretinoin

Tamoxifen, a selective modulator of estrogen receptor, is an effective first-line endocrine therapy that significantly improved relapse overall and relapse-free survival for many ER+ and endocrine-responsive patients. However, a significant proportion of the advanced ER+ BC patients do not respond (418). Recurrence occurs in approximately 40% of patients (419). As pinpointed in this subnetwork, seven miRNAs could sensitize cells to tamoxifen and might serve as potential therapeutic approaches for overcoming tamoxifen-resistance in BC: miR-27b, miR-375, miR-148a, miR-152, miR-206, miR-26a, miR-26b. Conversely, only three miRNAs confered tamoxifen resistance: miR-221, miR-222, miR-335. Lastly, aberrant expression of lncRNAs has also been linked to cancer progression and metastasis (56, 420). In the complex network of ER signaling, lncRNAs are emerging as critical determinants of hormone action. As opposed to miRNAs, high expression of lncRNAs, namely LINC-ROR (248, 249), MALAT1, CCAT2, was often associated with tamoxifen treatment failure in BC: their knock-down improved tamoxifen responsiveness in BC cells while uc.57 lncRNA promoted drug sensitivity. miR-27b had a different expression pattern between tamoxifen-sensitive vs. -resistant BC cell lines (421). In particular, miR-27b was found to be down-regulated in breast tumor tissues from tamoxifen-resistant patients (422) and high levels of miR-27b correlated with poor prognosis in BC (423, 424). CSC generation and EMT are essential events in tumor cell invasion and metastasis, both present in resistance to tamoxifen (425, 426). Of note, miRNAs have been associated with EMT and resistance to standard therapies. A direct target of miR-27b in modulating drug resistance and EMT is HMGB3 (427), an oncogene that can modulate drug resistance, proliferation and metastasis (428). Notably, while tamoxifen repressed miR-27b expression, estrogen induced miR-27b in BC cells (422). We already illustrated above miR-375, that can modulate the sensitivity/resistance of drug treatments in different cancers, including BC (193) and MTC (196). At the same time an anticancer treatment like alitretinoin may exert a regulatory action on miR-375 expression in BC cells (195). miR-148a and miR-152 reduced tamoxifen resistance in ER+BC via direct down-regulating the activated leukocyte cell adhesion molecule (ALCAM) (429). miR-206 was elevated in ER+BC cell lines (161) and its knock-down induced resistance to tamoxifen, while its overexpression reduced it by regulating G1/S-related proteins (430). miR-26a/b levels were lower in tamoxifen-resistant ER+BC and the inactivation of miR-26a/b decreased tamoxifen responsiveness of cancer cells (431). Additionally, miR-26 was found to be frequently downregulated in HCC and correlated with poor survival. miR-26b significantly suppressed the NF-κB signaling and dramatically enhanced chemo-sensitivity of HCC to doxorubicin by targeting TAK1 and TAB3, two positive regulators of NF-κB pathway (432). Subnetwork 10 also includes miR-221 and miR-222. These two miRNAs have a bivalent role in drug resistance across different cancer types. In this subnetwork miR-221/221 were found to enhance tamoxifen resistance (161, 163, 433). miR-335, promoted estrogen signaling, resulting in increased potency of tamoxifen. Additionally, tumor cells with acquired tamoxifen resistance did not show miR-335 nor ESR1 expression (434). The Metastasis associated in lung adenocarcinoma transcript 1 lncRNA (MALAT1), is over-expressed in several human malignancies, including ER+BC (435). High MALAT1 levels were also associated with tamoxifen treatment failure by regulating the transcription and splicing of ESR1, thus affecting ER signaling (436). Accordingly, MALAT1 may serve as an oncogenic lncRNA in pancreatic cancer, by promoting EMT, decreasing chemosensitivity to anticancer drugs and accelerating tumor angiogenesis (437). The CCAT2 lncRNA is overexpressed in BC, with the highest expression in lymph node negative patients. However, its expression levels are informative solely for a subgroup of patients, namely for lymph node positive patients that received adjuvant 5-fluorouracil, cyclophosphamide and methotrexate chemotherapy: high levels of CCAT2 suggested that patients would not benefit from CMF (438). Tamoxifen-resistant cells present a higher level of CCAT2 compared with sensitive cell, and knockdown of CCAT2 improved their response to tamoxifen (420). The levels of transcribed ultraconserved region uc.57 are lower in BC tissues than in precancerous breast tissues. uc.57 overexpression down-modulated BCL11A and reduced tamoxifen resistance in BC cells MCF7R by inhibiting the PI3K/AKT and MAPK signaling pathways (439).

### Subnetwork 11: imatinib

Imatinib (IM) is a 2-phenyl-amino-pyrimidine, an ATP-competitive tyrosine kinase inhibitor (TKI) and one of the most potent inhibitors of ABL1. Imatinib was approved for clinical treatment of CML but the problem of drug resistance encouraged the development of new TKI generations (440). Various ncRNAs have been associated with imatinib in CML, either as enhancers or inhibitors. HOX Antisense Intergenic RNA (HOTAIR) is located in the antisense strand of the HOXC gene locus, flanked by HOXC11 and HOXC12 (441). HOTAIR expression levels correlated with metastasis in BC and its loss was linked to decrease in invasion potential (442). HOTAIR lncRNA was up-regulated in CML patients with high levels of MDR1. Moreover, the knockdown of HOTAIR led to down-regulation of MDR1 resulting in higher sensitivity to imatinib; an involvement of HOTAIR in the PI3K/Akt pathway was also proposed (443). HULC is located at 6p24.3 and its transcript is a ~500 nt long, spliced and poly-adenylated lncRNA that localizes to the cytoplasm (444). The impact of HULC in hematologic malignancies is not clear yet, but it could act as a sponge for miRNA-372 in acute lymphoblastic leukemia (445). Moreover, HULC is involved in K562 cells survival and its silencing leads to increased apoptosis in CML cells by up-regulating PI3K/AKT signaling and c-Myc (446). Colorectal cancer, gastric cancer and melanoma show aberrant expression of SNHG5 (447, 448). SNHG5 lncRNA promotes imatinb resistance in CML and, although the mechanism may be complex, it seems to act as a competing endogenous RNA for miR-205-5p (449). UCA1 lncRNA located at 19p13.12, has an important role in drug resistance (114, 116). let-7i cooperates with miR-224 to revert imatinib resistance in CML (224). miR-1301 is involved in human cancers but shows an ambiguous behavior (450, 451). It can target the Ran GTPase Activating Protein 1 (RanGAP1) mRNA, as demonstrated by inverse correlation in CML patients: the RanGAP1 protein down-regulation or an increased miR-1301 are beneficial for the sensitivity to imatinib (452). miR-7 acts as an inhibitor in hepatocellular and pancreatic carcinomas (453, 454) possibly regulating the PI3K/AKT pathway, which is also downstream of BCR-ABL (455). In fact, over-expression of miR-7 in K562 cells, exhibit a significant inhibition of proliferation and increase of apoptosis via inhibition of BCR-ABL/PI3K/AKT signaling. Another report showed that miR-7 could work in synergy with imatinib to sensitize K562 (456). As the last ncRNA in this subnetwork, miR-518a is down-regulated in imatinib-resistant gastrointestinal stromal tumor (GIST) and PIK3C2A was identified as the relevant specific target (457).

### Non-connected RNA and drug nodes

Few ncRNA and drug combinations are not connected to the main network (and also obviously not to the subnetworks). One of these drugs is dactilosib, an imidazoquinoline derivative under phase II trial that works as dual inhibitor of PI3K and mTOR. It might improve conventional drug treatments and overcome some intrinsic adverse reactions of rapamycin and its derivates (458). Deng et al. studied dactilosib in AML and discovered that it caused up-regulation of miR-1-3p and consequent down-regulation of its targets involved in apoptosis, migration and multidrugs resistance. Moreover inhibition of miR-1-3p could interfere with dactilosib anti-proliferation effects (459).

In several human cancers miR-144 and miR-451 were identified as tumor suppressor ncRNAs (460). In terms of chemoresistance, miR-144 reversed 5-FU and imatinib resistance respectively in HCC (461) and leukemia (462). In addition, miR-144 might promote cisplatin sensitivity in prostate cancer (463) and in thyroid carcinoma (464). Whereas miR-144-3p contributed to sunitinib resistance in RCC by targeting ARID1A, a cancer gene involved in chromatin remodeling (465). Reduction of ARID1A expression could also serve as a predictive biomarker for trastuzumab resistance in BC (466). Although breast and ovarian cancer have comparable levels of HER2/ErbB2 expression patterns, pertuzumab treatment is more effective in BC. Wuerkenbieke et al. investigated this effect and found miR-150 knockdown in ovarian cancer; this might contribute to enhance pertuzumab resistance (467). ncRNAs can also be related to side-effects occurring upon cancer treatment: vascular events are a serious problem in CML patients treated with tyrosine kinase inhibitors like nilotinib. Recent findings suggest that nilotinib decreases levels of miR-3121-3p resulting in higher levels of IL-1β and adhesion molecules in vascular endothelial cells. (468). miR-132-5p expression, via CYP1A2 modulation, could reduce flutamide-induced hepatic cell toxicity (469). Finally, in a matrix *in vitro* screen of several miRNAs and drugs in BC, miR-126 augmented the potency of CDK4/6 or PIK3CA inhibitors in MCF7 (Luminal) and MDA-MB-453 (HER2^+^) cell lines (470).

## Author contributions

SV contributed to design and critical revision. FC, LM, and FB performed collection and curation of data. FC assembled data and wrote the article. LM and FB contributed to network analysis. CA contributed to network analysis and writing. All authors read and approved the final manuscript.

### Conflict of interest statement

The authors declare that the research was conducted in the absence of any commercial or financial relationships that could be construed as a potential conflict of interest.

## Supplementary material

Supplementary Image 1 The complete ncRNA/drug network. The figure illustrates the connected network of all drug/ncRNA interactions (edges) and 5 unconnected pairs. The network represents a human curated selection of papers listed in PubMed to identify the most important ncRNAs/cancer drugs interactions and cliques.

Supplementary Table 1 ncRNA/drug network indices. The Excel file contains the measures for all nodes (drugs/ncRNAs) in the network.

Supplementary Table 2 ncRNA/drug interaction effect on chemoresistance. This table shows, for each subnetwork, the non-coding RNAs that influenced chemoresistance, or drug sensivity, as reported in our review. Red represents inhibition, and green enhancement, of the relative process. We indicated all drugs in the subnetworks and the related ncRNAs behaviors. Keys: ↑ ncRNA up-regulation, ↓ ncRNA down-regulation.
