# Supplementary figures and images for: The Network of Non-coding RNAs in Cancer Drug Resistance
Source: Front Oncol. 2018 Aug 29;8:327. doi: 10.3389/fonc.2018.00327 (PMC6123370; doi:10.3389/fonc.2018.00327)

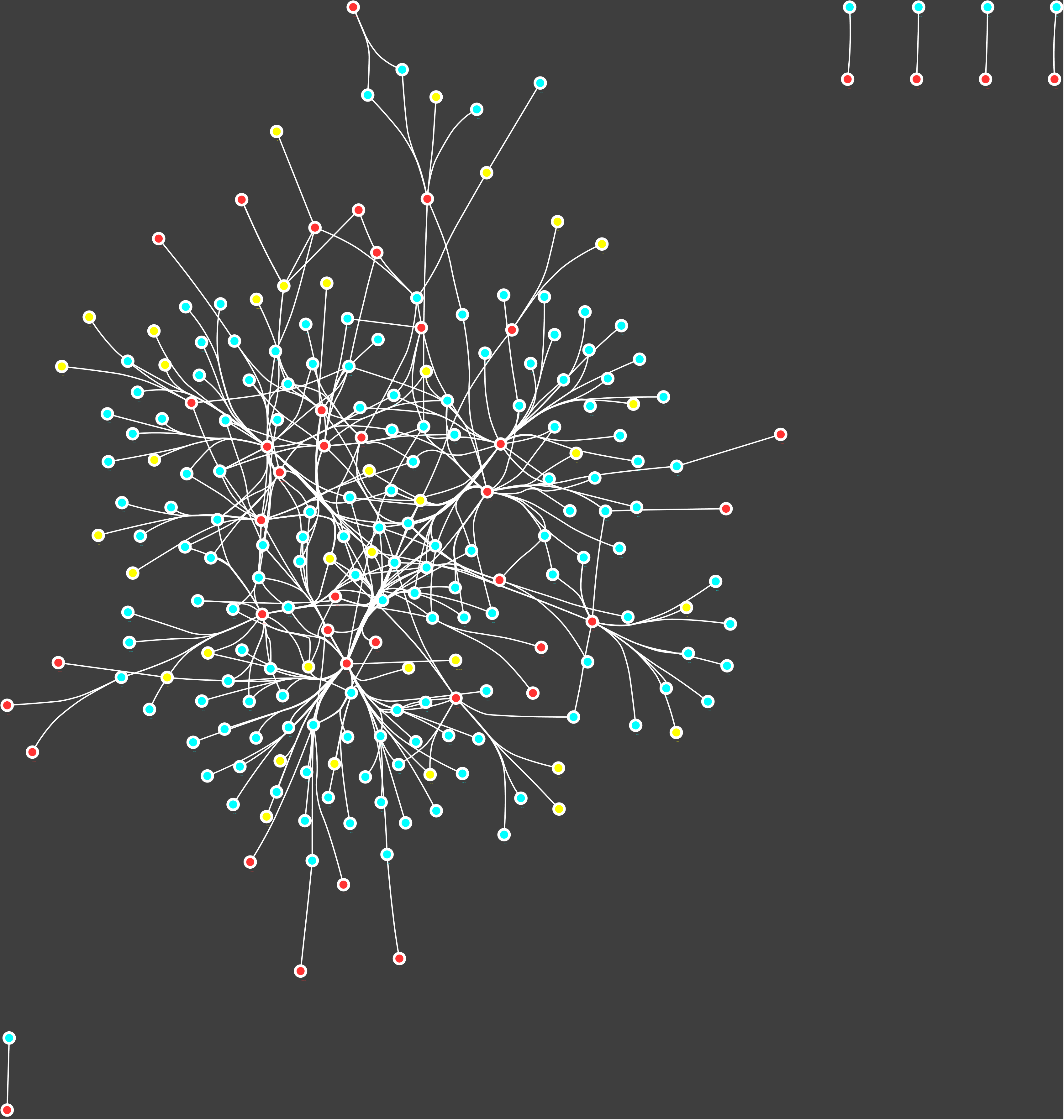

Supplement: Supplementary Image 1 — The complete ncRNA/drug network. The figure illustrates the connected network of all drug/ncRNA interactions (edges) and 5 unconnected pairs. The network represents a human curated selection of papers listed in PubMed to identify the most important ncRNAs/cancer drugs interactions and cliques. [file Image_1.TIFF]
